# Prospective comparison of D1 *vs* modified D2 gastrectomy for carcinoma

**DOI:** 10.1038/sj.bjc.6601790

**Published:** 2004-05-04

**Authors:** P Edwards, G R J C Blackshaw, W G Lewis, J D Barry, M C Allison, D R B Jones

**Affiliations:** 1 Department of Surgery, Royal Gwent Hospital, Cardiff Road, Newport NP20 2UB, UK; 2 Department of Gastroenterology, Royal Gwent Hospital, Cardiff Road, Newport NP20 2UB, UK; 3 Department of Surgery, Nevill Hall Hospital, Brecon Road, Abergavenny NP7 7EG, UK

**Keywords:** Gastric cancer, Surgery, Lymphadenectomy

## Abstract

To compare the outcomes after D1 gastrectomy with those after modified D2 gastrectomy (preserving pancreas and spleen) performed by specialist surgeons for gastric cancer in a large UK NHS Trust. In all, 118 consecutive patients with gastric adenocarcinoma were referred by postcode, to undergo either a D1 gastrectomy (North Gwent (RJ), *n*=36, median age 76 years, 21 m) or a modified D2 gastrectomy (South Gwent (WL), *n*=82, 70 years, 57 m). Operative mortality in the two groups of patients was similar (D1 8.3% *vs* D2 7.3%, *χ*^2^ 0.286, DF 1, *P*=0.593). Overall cumulative survival at 5 years was 32% after D1 gastrectomy compared to 59% after D2 gastrectomy (*χ*^2^ 4.25, DF 1, *P*=0.0392). In patients with stage III cancers, survival was 8% after D1, compared with 33% after D2 gastrectomy (*χ*^2^ 6.43, DF 1, *P*=0.0112). In a multivariate analysis, T stage (hazard ratio 2.339, 95% CI 1.683–2.995, *P*=0.01), N stage (hazard ratio 4.026, 95% CI 3.536–4.516, *P*=0.0001) and the extent of lymphadenectomy (hazard ratio 0.258, 95% CI –0.426–0.942, *P*=0.0001) were independently associated with durations of survival. In conclusion, modified D2 gastrectomy can improve survival four-fold for patients with stage III gastric cancer, without significantly increasing morbidity and mortality when compared with a D1 gastrectomy.

Opinion over the optimum resection for patients with gastric cancer remains divided, and the literature polarised. The impressive outcomes after D2 gastrectomy published in large retrospective series from Japan (Soga *et al*, 1979; Maruyama *et al*, 1987) have not been reproduced in randomised comparative studies from Europe ([Bibr bib8][Bibr bib7]; [Bibr bib10][Bibr bib11]). The two largest randomised studies both report significantly greater operative morbidity and mortality associated with an extended D2 lymphadenectomy when compared with the less aggressive D1 lymphadenectomy, and have failed to demonstrate any survival advantage for a D2 resection. Many of the serious complications associated with D2 resections were associated with resections of the pancreas and spleen (Bonenkamp *et al*, 1995; Cuschieri *et al*, 1996), and the best long-term survival was observed in patients undergoing D2 gastrectomy without pancreatico-splenectomy (Cuschieri *et al*, 1999). Although this latter report concluded than a classical D2 resection offered no survival advantage over a D1 resection, the possibility that a modified D2 resection, preserving pancreas and spleen, might be better than a D1 resection was not dismissed (Cuschieri *et al*, 1999).

The first reports of outcomes after modified D2 gastrectomy for gastric cancer were originally published in Britain by Sue-Ling *et al*, 1993 (Sue-Ling *et al*, 1993) and subsequently Griffith *et al* (1995) from the University Department of Surgery at Leeds. We have subsequently demonstrated that similar results can be achieved in a large British district general hospital (Lewis *et al*, 2002; Barry *et al*, 2003). Despite these favourable reports, there remains a widely held assumption, that poor outcomes after surgery for gastric cancer in Britain are due to the greater age, comorbidity, advanced stages of disease and greater body mass indices of Western patients when compared with their Japanese counterparts (Dhar *et al*, 2000). Furthermore, most oesophagogastric cancer surgery in Britain and much of the West, has by tradition, been performed by general surgeons. Radical lymphadenectomy is a painstaking, technically demanding procedure, which has usually remained within the province of small numbers of specialist upper gastrointestinal surgeons (Roder *et al*, 1993). Thus, the D1 perigastric lymphadenectomy remains the most commonly performed operation for gastric cancer in the West.

The aim of our study was to investigate whether a modified D2 gastrectomy, allied to a specialist multidisciplinary team approach, would improve outcomes when compared with the traditional D1 gastrectomy. The setting was a large British NHS Trust comprising two District General Hospitals in South Wales serving a total population of 560 000.

## PATIENTS AND METHODS

Between January 1996 and December 2002, 429 consecutive patients with adenocarcinoma of the stomach were treated by two consultant general surgeons with subspecialist interests in upper gastrointestinal surgery in Gwent, South Wales. Patients enrolled into the study were to have histologically proven, and potentially curable, gastric carcinoma. Patients were excluded if they had undergone previous gastric surgery, or had serious comorbidity that would preclude a safe D2 gastrectomy. All patients underwent staging laparoscopy to define potentially curable disease. Eligible patients were those who fell within the UICC TNM cancer stages I to III (Kennedy, 1987). Patients with North Gwent postcodes (catchment population 110 000) were treated at Nevill Hall Hospital, Abergavenny (DRBJ), and those with South Gwent postcodes (catchment population 450 000) were treated at the Royal Gwent Hospital, Newport (WGL).

Clinical and pathological information was collected prospectively and indices of multiple deprivation (IMD) were obtained from the Office for National Statistics. In total, 164 patients underwent treatment at Nevill Hall Hospital. Potentially curative resection was possible in 36 out of the 164 patients treated at Nevill Hall Hospital (22%). The median age of these 36 patients was 77 (range 58–93) years and 22 were male. This comprises our control group (D1). In all, 265 patients underwent treatment at the Royal Gwent Hospital. Potentially curative resection was possible in 82 (31%, *χ*^2^ 4.108, DF 1, *P*=0.043). The median age of this group of patients was 70 (range 27–86) years (*P*=0.0001) and 57 were male. This comprises our comparison group (D2). The definition of a potentially curative resection was that all visible tumour was removed and that both proximal and distal resection margins were free of tumour on histological examination. Ethical approval was granted by the Gwent Research Ethics Committee.

### Surgical treatment

The entire surgical management was conducted by two consultant surgeons working independently in the two major district general hospitals within Gwent Healthcare NHS Trust (DRBJ, Nevill Hall Hospital, North Gwent, and WGL, Royal Gwent Hospital, South Gwent). The operative details of the patients were defined in terms of the extent of the gastric resection, the macroscopic tumour-free margins and the level of the lymphadenectomy. Preoperative staging was done with the aid of both spiral computerised tomography and laparoscopy. All tumours were staged in accordance with the 1987 Unified Classification of gastric cancer (Kennedy, 1987), until 1997, when we adopted the recently published TNM Classification of Malignant Tumours (Sobin and Wittekind, 1997). The details of the patients undergoing potentially curative resection together with their ASA grades and stages of disease are shown in Tables 1

**Table 1:** Details of the patients related to survival

	**D1 surgery**		**D2 surgery**	
	***n***	**5-year survival (%)**	***n***	**5-year survival (%)**
Number	36		82	
Sex				
m	21 (58)	43	57 (70)	62
f	15 (42)	24	25 (30)	53
				
Age (years)				
<60	3 (8)	100	13 (16)	78
60–69	9 (25)	50	27 (33)	62
70+	24 (67)	36	42 (51)	45
				
ASA				
I	4 (11)	67	6 (7)	100
II	11 (30)	27	38 (46)	66
III	21 (59)	30	38 (47)	50
				
Location				
c	5 (14)	0*	22 (26)	63*
m	5 (14)	n/a	17 (21)	62
a	25 (69)	26	40 (49)	57
c,m,a	1 (3)	0	3 (4)	34
				
Sx				
Yes	3 (8)	0	7 (9)	43
No	33 (92)	33**	75 (91)	60**
				
Px				
Yes	0	n/a	3 (4)	66
No	0	n/a	79 (96)	58

Figures are numbers of patients, percentages in parentheses. c=cardia, m=mid (body), a=antrum, cma=linitis plastica, Sx=splenectomy, Px=pancreatectomy.

* *P*<0.003

** *P*<0.05.

and 2

**Table 2:** Details of the stages of disease related to survival

	**D1 surgery**		**D2 surgery**	
	***n***	**5-year survival (%)**	***n***	**5-year survival (%)**
T stage				
T1	7 (19)	100	7 (8)	100
T2	9 (25)	82	13 (16)	86
T3	19 (53)	17*	48 (59)	51*
T4	1 (3)	n/a	14 (17)	36
				
N stage				
N0	16 (44)	56**	38 (46)	92**
N1	15 (42)	14**	24 (29)	54**
N2	4 (14)	n/a	20 (24)	n/a
				
Stage				
I	12 (33)	70	16 (20)	80
II	9 (25)	100	23 (28)	96
IIIA	12 (33)	10†	19 (23)	68†
IIIB	3 (8)	n/a	24 (29)	5

Figures are numbers of patients, percentages in parentheses.

* *P*=0.006

** *P*<0.05

† *P*=0.0003.

. The details of their surgery are shown in Table 3

**Table 3:** Details of the surgery in patients undergoing a R0 resection

	**D1 (*n*=36)**	**D2 (*n*=82)**
**Operation**	***n***	***n***
Subtotal gastrectomy	28	47*
Total gastrectomy	8	35*
Splenectomy	3	7
Distal pancreatectomy + Sx	0	3
Transverse colectomy	0	11**

Figures are numbers of patients.

* *P*=0.03

** *P*=0.02. Sx=splenectomy.

.

D1 resections entailed removal of the lymph nodes within 3.0 cm of the tumour *en bloc* with the greater omentum and stomach. D2 resections necessitated the additional removal of the omental bursa, the hepatoduodenal nodes (antral tumours) and the splenic artery nodes. In patients with tumours of the posterior proximal stomach invading the tail of pancreas or splenic hilum, a splenectomy and distal pancreatectomy were also performed. In both groups of patients, a distal gastrectomy up to and including the duodenal bulb with a minimum of 5 cm proximal tumour-free margin was performed for antral tumours, whereas total gastrectomy was performed for middle and proximal tumours.

### Follow-up

Patients undergoing both D1 and D2 resections were reviewed every 3 months for the first year and every 6 months thereafter. Only one of the 118 patients was lost to follow-up and 80 patients were followed up for a minimum of 5 years or until death (24 D1, 56 D2). The median duration of follow-up was 36 months. Endoscopy and computed tomography were arranged if recurrent disease was suspected. Causes of death were sought from case notes, general practitioners’ records or via the Office for National Statistics.

### Statistical analysis

Sample size calculations were based on a prestudy literature survey of six studies, which indicated that the baseline 5-year survival rate of D1 surgery was expected to be 20%, and improvement in survival to 60% with D2 resection would be a realistic expectation. Thus, 70 patients (30 in each arm) were to be studied, providing 90% power to detect such a difference with *P*<0.05. The calculations of statistical power were performed with the ‘nQuery Advisor’ statistics package (Statistical Solutions, Stonehill Corporate Center, Suite 104, 999 Broadway, Saugus, MA 01906, USA). The analysis of the results has been on an intention-to-treat basis. Statistical analysis appropriate for non parametric data was used. Grouped data were expressed as median (interquartile range). Groups were compared with the Mann–Whitney *U*-test for unpaired data. Nominal data were analysed by means of Fisher's exact test (Altman, 1991). Cumulative survival was calculated by the life-table method of Kaplan and Meier (1958). Differences in survival times between groups of patients were analysed by the log-rank method (Peto *et al*, 1977). Cox's proportional hazards model was used to fit the multivariate survival model. Data analysis was carried out with the Statistical Package for Social Sciences (SPSS) version 11 (SPSS, Chicago, IL, USA).

## RESULTS

### Stages of disease at presentation (Table 1)

There was no significant difference in the stages of disease in the two groups of patients, although the proportion of patients with stage I and II cancers was greater in patients undergoing D1 gastrectomy (58%) in North Gwent when compared with the patients undergoing D2 gastrectomy (48%) in South Gwent (*χ*^2^ 1.162, DF 1, *P*=0.281).

### Indices of multiple deprivation

The median IMD score in patients undergoing D1 gastrectomy in North Gwent was 32.08 (10.02–39.44) compared to 19.24 (8.44–29.65) in patients undergoing D2 gastrectomy in South Gwent (*P*=0.111).

### Operative morbidity and mortality

There was no significant difference between the ASA grades of the patients undergoing potentially curative surgery in the D1 group (ASA I, II, III, IV: 4, 11, 21, 0) compared to the patients undergoing potentially curative surgery in the D2 group (ASA I, II, III, IV: 6, 39, 37, 0). Operative morbidity was 25% after D1 gastrectomy compared with 23% after D2 gastrectomy (*χ*^2^=0.046, DF 1, *P*=0.830). Details of the major operative morbidity are shown in Table 4

**Table 4:** Details of the operative morbidity and mortality

	**D1**	**D2**
Anastomotic leak	3 (2)	2 (1)
Respiratory sepsis	2	9 (2)
Wound infection	0	3
Thromboembolic	1	2 (1)
Myocardial infarction	0	2 (2)
Stroke	1 (1)	1
Subphrenic collection	2	0
Total morbidity	9 (3)	19 (6)

Figures are numbers of patients. Operative deaths are in parentheses.

. Operative mortality 30 days after operation was 8.3% (three out of 36 patients) after D1 gastrectomy compared with 7.3% overall (six out of 82 patients) after a modified D2 gastrectomy (*χ*^2^=0.037, DF 1, *P*=0.848). None of the patients who died after a D2 gastrectomy had undergone a splenectomy or pancreatectomy.

### Learning curve

Operative mortality after D2 gastrectomy was considerably lower for the last 42 patients (2.4%) than for the first 40 patients (12.5%), though this decrease was not statistically significant (*χ*^2^=3.093,. DF 1, *P*=0.079).

### Lymph node sampling

The median number of lymph nodes sampled were eight (range 1–24) after D1 lymphadenectomy compared with 15 (range 5–32) after D2 lymphadenectomy (*P*=0.022).

### Survival

**Figure 1:**
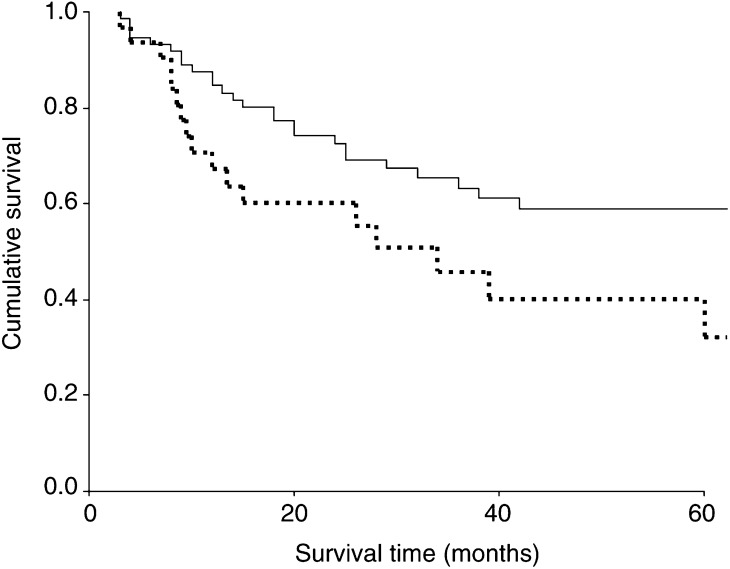
Corrected survival curves for all patients undergoing R0 gastrectomy. Log-rank *χ*^2^=4.25, DF 1, *P*=0.0392. Operative deaths were excluded. — D2 gastrectomy, - - - - - - D1 gastrectomy.

Corrected cumulative survival by treatment, calculated by life-table analysis, is shown in Figure 1

**Figure 2:**
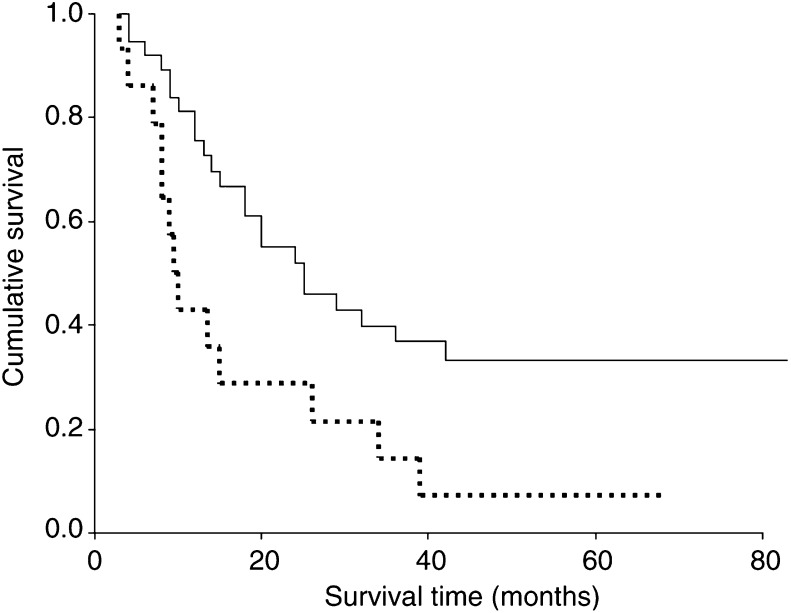
Corrected survival curves for patients with stage III gastric cancer undergoing R0 gastrectomy. Log rank *χ*^2^=6.43, DF 1, *P*=0.0112. Operative deaths were excluded — D2 gastrectomy, - - - - - - D1 gastrectomy.

. Cumulative survival for the 36 patients undergoing a D1 gastrectomy was 32% at 5 years. In contrast, survival for the 82 patients undergoing a D2 gastrectomy was 59% at 5 years (*χ*^2^ 4.25, DF 1, *P*=0.0392). For patients with stage I disease the cumulative survival for the patients undergoing a D1 gastrectomy was 70% at 5 years compared with 80% for the patients undergoing a D2 gastrectomy (*χ*^2^ 1.78, DF 1, *P*=0.1817). For patients with stage III disease the cumulative survival for the patients undergoing a D1 gastrectomy was 8% at 5 years compared with 33% for the patients undergoing a D2 gastrectomy (*χ*^2^ 6.43, DF 1, *P*=0.0112) Figure 2

. The factors found to be significantly associated with the duration of survival on univariate analysis are shown in Table 5

**Table 5:** Univariate analysis of factors associated with duration of survival.

	**All stages**		**Stage III**	
**Factor**	***χ*^2^**	***P***	***χ*^2^**	***P***
Pancreatectomy	0.16	0.6904	1.48	0.2235
Type of gastrectomy	0.29	0.5912	0.19	0.6662
Site of tumour	0.72	0.8682	1.56	0.6686
Gender	0.92	0.3375	0.09	0.7653
Splenectomy	1.72	0.1900	0.00	0.9837
Age	2.90	0.2342	1.70	0.4283
Lymphadenectomy	4.25	0.0392	6.43	0.0112
ASA grade	11.52	0.0213	4.17	0.3835
T stage	14.05	0.0071	0.02	0.8977
N stage	100.07	0.00001	12.11	0.0023
Stage of disease	108.74	0.00001	12.47	0.0004

.

### Multivariate analysis

The prognostic variables entered into the model were age, sex, ASA grade, stage of the tumour, location of the tumour, type of gastrectomy (subtotal or total), level of lymphadenectomy, and level of the resection of the spleen and pancreas. Forward and backward stepwise regression was used. T stage of disease (hazard ratio 2.339, 95% CI 1.683–2.995, *P*=0.01), N stage of disease (hazard ratio 4.026, 95% CI 3.536–4.516, *P*=0.0001) and extent of lymphadenectomy (hazard ratio 0.258, 95% CI –0.426–0.942, *P*=0.0001) were found to be the most important predictors of outcome as determined by Cox's proportional hazards model (global *χ*^2^ for the model was 67.494, DF 8, *P*=0.0001). For patients with stage III disease extent of lymphadenectomy (hazard ratio 0.249, 95% CI −0.517–1.015, *P*=0.0001) and stage of disease (hazard ratio 2.203, 95% CI 1.819–2.587, *P*=0.0001) were significantly and independently associated with duration of survival.

## DISCUSSION

Gastric cancer continues to be described as one of the ‘captains of the men of death’ in a major western text (Bailey and Love, 2000). The findings of this study demonstrate that gastrectomy with extended D2 lymphadenectomy, originally described by Japanese surgeons (Soga *et al*, 1979; Maruyama *et al*, 1987), can be introduced safely into a British Cancer Unit and may significantly improve the outcome for operable gastric cancer. In particular, our modified D2 gastrectomy (with preservation of the spleen) was associated with a three-fold improvement in survival for patients with stage III gastric cancer compared with a contemporaneous group of stage III patients undergoing a traditional D1 gastrectomy. Moreover, the proportion of patients for whom potentially curative resection was possible was significantly greater in patients undergoing a D2 gastrectomy compared to patients undergoing a D1 gastrectomy.

There are several potential criticisms of this study. Clearly, this is not a randomised trial, because patients were referred to the relevant surgeon according to their postal code. This may introduce two confounding variables; in studies of breast cancer and colorectal cancer postcode addresses of patients have been associated with survival differences, perhaps because social deprivation is commoner in certain postal districts (Sainsbury *et al*, 1995, Brewster and Black, 1998). Secondly, the postal address of each patient determined which one of two surgeons performed their operation. It is well recognised that the identity of the surgeon performing the operation is an independent determinant of stage-related survival (MacDonald *et al*, 2001). Yet the improved outcomes after D2 gastrectomy cannot be explained by unexpectedly poor results in our control group because outcome after D1 gastrectomy compared favourably with that reported in a large UK population-based study (Allum *et al*, 1989). In this latter study, less than 1% of patients had stage I disease (compared with 7% from our study and 18% from the MRC ST01 trial), and curative resection was possible in only 25% of patients (compared to 21% in the present study). Operative mortality was 16% after potentially curative D1 resection (compared to 8.3% in our study and 6.5% from the MRC ST01 trial) and 5-year survival was 20% (compared to 38% in our study and 35% from the MRC ST01 trial). The surgical management of patients with gastric cancer at Nevill Hall Hospital was therefore at least as good as that of most other centres performing D1 gastrectomies in the United Kingdom.

The reluctance of Western surgeons to adopt extended lymphadenectomy is understandable in the light of the results of the MRC ST01 trial of D1 *vs* D2 resection for gastric cancer ([Bibr bib10][Bibr bib11]) and the Dutch study ([Bibr bib8][Bibr bib7]). Each reported similar mortality following D2 gastrectomy (13 and 10%, respectively), and both include evidence to suggest that the excess mortality is accounted for by splenectomy (with or without the resection of the pancreas) rather than the extended lymphadenectomy. Moreover, in MRC ST01 the best survival was obtained in patients who underwent D2 resections without pancreatico-splenectomy. The incidence of lymph node metastases along the splenic artery and splenic hilum is reported to vary from 15 to 27% (Fass and Schumpelick, 1989; Mendes de Almedida *et al*, 1995; Tsubaraya *et al*, 1995). Many of these nodes can be cleared with preservation of the pancreas and spleen. D2 gastrectomy with preservation of the spleen has already been shown to confer potential survival benefits with low morbidity and mortality (Griffith *et al*, 1995).

Both MRC ST01 and the Dutch trial have received criticism over the relative inexperience of many different surgeons performing D2 lymphadenectomy. Furthermore, the existence of a relationship between caseload and operative mortality remains controversial. The specialist surgical unit in Leeds has described a long learning curve during the adoption of D2 gastrectomy, with 10 years elapsing before operative mortality fell to 5% (Sue-Ling *et al*, 1993). Our results parallel this finding, with surgical subspecialisation reducing operative mortality after D2 gastrectomy from 12.5 to 2.4% over a period of 5 years. Operative mortality fell during this period even though there were no differences in stage of cancers at presentation or postoperative complications. This serves to emphasise that low operative mortality is achieved not only by specialised operative technique, but also by specialised anaesthetic and postoperative care from an experienced multidisciplinary team.

In conclusion, this nonrandomised study points to potential survival benefits arising from modified D2 gastrectomy in patients with gastric cancer of pathological stage T3 NX M0. The results strongly support the establishment of a multicentre randomised controlled trial of modified D2 *vs* D1 gastrectomy, each performed by expert gastric surgeons and targeted towards patients with tumours of intermediate stage. The practical problem that we face in Britain is that most patients present with advanced stage III and IV cancers, and preoperative staging modalities remain of limited sensitivity (Daly *et al*, 1999; Barry *et al*, 2002). Moreover, additional confounding variables, such as surgical subspecialisation and multi disciplinary team learning curves may result in treatment contamination and noncompliance, to the extent that implementation would require robust quality control (McCulloch *et al*, 2003). Stage-directed management tailored to individual patients is clearly the way forward if the benefits of greater subspecialisation and a multidisciplinary team approach are to be realised (Furukawa *et al*, 2002). The exact role of D2 gastrectomy following neoadjuvant chemotherapy (Allum *et al*, 2003) or prior to chemoradiotherapy (MacDonald *et al*, 2001) remains unclear at present, but our own experience is encouraging and suggests that in otherwise fit patients with gastric tumours that are perceived to be of stage III, without distant metastatic disease, a modified D2 gastrectomy preserving pancreas and spleen where possible, and performed by specialist surgeons, may have advantages over the traditional standard D1 gastrectomy.
